# Predictors of imminent risk of fracture in Medicare-enrolled men and women

**DOI:** 10.1007/s11657-020-00784-7

**Published:** 2020-08-03

**Authors:** Akeem A. Yusuf, Yan Hu, David Chandler, Daria B. Crittenden, Richard L. Barron

**Affiliations:** 1 Amgen Inc., Thousand Oaks, CA USA; 2 Boston Scientific Corporation, Arden Hills, MN USA

**Keywords:** Imminent risk, Near-term risk, Fracture, Osteoporosis, Medicare

## Abstract

**Summary:**

Advancing age, female sex, recent prior fracture and falls, and specific comorbidities and medications contribute to imminent (within 1–2 years) risk of fracture in Medicare enrollees. Clinician awareness of these risk factors and their dynamic nature may lead to improved osteoporosis care for elderly patients.

**Purpose:**

The burden of osteoporotic fracture disproportionately affects the elderly. Growing awareness that fracture risk can change substantially over time underscores the need to understand risk factors for imminent (within 1–2 years) fracture. This study assessed predictors of imminent risk of fracture in the US Medicare population.

**Methods:**

Administrative claims data from a random sample of Medicare beneficiaries were analyzed for patients aged ≥ 67 years on January 1, 2011 (index date), with continuous coverage between January 1, 2009 and March 31, 2011, excluding patients with non-melanoma cancer or Paget’s disease. Incident osteoporotic fractures were identified during 12 and 24 months post-index. Potential predictors were age, sex, race, history of fracture, history of falls, presence of osteoporosis, cardiovascular diseases, chronic obstructive pulmonary disorder (COPD), mood/anxiety disorders, polyinflammatory conditions, difficulty walking, use of durable medical equipment, ambulance/life support, and pre-index use of osteoporosis medications, steroids, or central nervous system medications. Cox proportional hazards models were used to evaluate predictors of fracture risk in the two follow-up intervals.

**Results:**

Among 1,780,451 individuals included (mean age 77.7 years, 66% female), 8.3% had prior fracture and 6.1% had a history of falls. During the 12- and 24-month follow-up periods, 3.0% and 5.4% of patients had an incident osteoporotic fracture, respectively. Imminent risk of fracture increased with older age (double/triple), female sex (> 80%), recent prior fracture (> double) and falls, and specific comorbidities and medications.

**Conclusions:**

Demographics and factors including fall/fracture history, comorbidities, and medications contribute to imminent risk of fracture in elderly patients.

**Electronic supplementary material:**

The online version of this article (10.1007/s11657-020-00784-7) contains supplementary material, which is available to authorized users.

## Introduction

The risk of osteoporotic fracture increases with age, and the direct cost of treating osteoporosis-related fractures is expected to increase substantially as the number of incident fractures increases, especially in more economically developed countries [1–3]. In both private (commercial) and public (Medicaid, Medicare) health plans in the United States (US), patients with osteoporosis-related fracture have been shown to have higher medical costs than individuals without fractures [4, 5]. Although osteoporosis patients may range in age, the literature suggests that burden of osteoporosis is concentrated among the elderly, in terms of both fracture incidence and fracture-related costs. One retrospective study, for example, found that 70% of fractures and 87% of costs were incurred by Americans who were 65 years and older (thus eligible for Medicare), and that inpatient stays and long-term care were the largest contributors to total osteoporosis costs [1]. In the US, the total direct cost of incident and prevalent osteoporosis-related fractures is projected to increase from $19 billion in 2005 to $25.3 billion in 2025, with this growth largely driven by fracture incidence among Medicare-aged individuals [1]. In addition to this direct cost burden, osteoporosis-related fractures of all types also may result in lasting impairments to quality of life, mobility, social and physical function, and increased risk of mortality lasting up to 5 years after a vertebral fracture and up to 10 years after a hip fracture [6, 7].

Fracture risk assessment tools have been incorporated into clinical guidelines in an effort to identify individuals for osteoporosis management, including pharmacologic treatment to prevent fractures, untimely death, and to reduce osteoporosis-related costs to patients and society [8]. FRAX®, the most widely available such tool, uses patient demographic and clinical characteristics to estimate fracture risk over a 10-year time horizon. While this long-term risk perspective is valuable for identifying patients for osteoporosis therapy, these tools may only be used once for a given patient, an approach that does not acknowledge the increasing appreciation that fracture risk is not always linear [9, 10]. For example, medical events such as stroke or myocardial infarction and the use of certain types of medications may have both immediate and lasting effects on patients’ physical functioning, balance, or gait which, in turn, may increase the risk of falling, a significant fracture risk factor [11]. Coincident with this growing awareness of the dynamic nature of fracture risk, research identifying specific factors contributing to imminent risk of fractures (i.e., within the next 1–2 years) is accumulating [10, 12–15]. As this field of research expands, the data obtained may be used to determine an appropriate threshold of risk that is clinically meaningful (e.g., percent of patients expected to experience a fracture within the next year) and can be used as a basis for treatment recommendations.

Given the greater incidence of fracture and more significant clinical, functional, quality of life, and cost consequences in the elderly, the current study was designed to identify predictors of imminent risk of fracture in the US Medicare population.

## Methods

This study was conducted with data from inpatient, outpatient, and prescription drug claims in the 20% Medicare database, which includes information for a random sample of beneficiaries covered by Medicare Parts A, B, and D. The Part A and Part B database contain data on patient enrollment, clinical utilization, and expenditures for inpatient and outpatient professional and facility-based care as well as skilled nursing facilities and hospice care. The Medicare Part D database contains details of prescription drug utilization and costs. Medicare beneficiaries included in this analysis were aged ≥ 67 years on January 1, 2011 (index date). To ensure complete capture of data on important covariates in the period prior to the index date, study patients were continuously enrolled in Medicare Parts A, B, and D between January 1, 2009 and March 31, 2011, and individuals with participation in any health maintenance organization (Medicare Advantage) plan were excluded. No other coverage-type exclusions were made. To reduce the risk of misclassification and increase the likelihood that any observed fractures were related to osteoporosis, patients with evidence of malignant neoplasm (excluding melanoma) or Paget’s disease of bone in the 12 months prior to the index date were also excluded.

All patients were required to have 24 months of continuous plan enrollment prior to the index date. Patients were followed for up to 24 months after the index date and the occurrence of incident osteoporotic fracture was assessed during 12- and 24-month follow-up intervals. Patients who contributed data to each assessment interval were required to have continuous plan enrollment during that interval. These fractures were identified with a published algorithm designed for use with administrative claims data [16]. Fractures were indicated by the presence of either (1) at least one inpatient claim with a relevant International Classification of Diseases, Ninth Revision, Clinical Modification (ICD-9-CM) diagnosis code or a Common Procedure Terminology (CPT) fracture repair procedure code or (2) an outpatient claim with both a qualifying diagnosis code and a fracture-related procedure code. Fractures with major trauma codes (E-codes) recorded concurrently were excluded from analysis.

The pool of potential predictors of imminent fracture risk was based on the existing literature on fracture risk factors, including recent studies examining factors associated with imminent risk of fracture. Predictor variables considered in this study included demographics (age, gender, race), history of fracture, history of falls, comorbid conditions (osteoporosis, cardiovascular diseases, chronic obstructive pulmonary disorder [COPD], mood and anxiety disorders, polyinflammatory conditions [rheumatoid arthritis, ankylosing spondylitis, osteoarthritis]), variables that may be markers for frailty (use of durable medical equipment, ambulance/life support, difficulty walking, paralysis, weakness, podiatric care), and baseline osteoporosis medication use and use of medications associated with secondary osteoporosis and/or fall risk (corticosteroids, central nervous system medications, antihypertensives). With the exception of fracture history which was determined during 24 months prior to the index date, these variables were quantified during a 12-month pre-index period.

Multivariate Cox proportional hazards models were used to evaluate potential predictors of imminent risk of fracture, with separate models constructed to identify predictors of fracture risk in 12 months and 24 months of follow-up. In the initial analysis, the models included all potential predictors. Reduced, parsimonious models were obtained using the augmented backward elimination algorithm with a 10% change in estimate criterion required to retain a variable in the final model [17]. A risk-scoring point system based on the Sullivan methodology [18] was applied to convert the model results into risk scores. With this methodology, increasing numbers of points were assigned to increasing levels of risk (e.g., increasing age was associated with a greater number of points), and the totaled points correspond to an individual’s predicted fracture probability during follow-up. All analyses were conducted using SAS® software, version 9.2 (SAS Institute Inc., Cary, NC).

## Results

Baseline demographic, clinical, and healthcare utilization characteristics for the 1,780,451 individuals who met all study inclusion criteria are summarized in Table 1. The mean (standard deviation [SD]) age of patients in the study cohort was 77.7 years (7.5); 66.0% of patients were female and 85.2% of patients were white. In the 24 months prior to the index date, 8.3% of patients had experienced a fracture and 6.1% had a history of falls.

**Table 1:** Distribution of selected demographic, clinical and other factors in the study cohort

Characteristic	Mean (SD)/Percent
Total patients	1,780,451
Demographics	
Age in years, mean (SD)	77.7 (7.5)
Male, percent	34.0
White, percent	85.2
Selected clinical characteristics, percent	
Hospitalized	18.9
History of fracture	8.3
History of falls	6.1
Osteoporosis	5.5
Congestive heart failure	9.1
Diabetes mellitus	25.7
COPD	12.5
Gastrointestinal disorders/toxicities	6.5
Chronic kidney disease	8.7
Anemia	13.5
Thyroid disease	12.1
Rheumatoid arthritis	2.0
Osteoarthritis	13.7
Depression	6.5
Dementia	7.4
Mood or anxiety disorders	3.6
Ankylosing spondylitis	< 1
Frailty markers, percent	
Use of DME	11.3
Ambulance/life support	14.7
Difficulty walking	15.4
Paralysis	2.5
Weakness	10.6
Podiatric care	11.9
Mitigating factors, percent	
Physical occupational therapy	23.7
Nurse home services	6.2
Home healthcare services	10.7
Concomitant medications, percent	
Osteoporosis medications^a^	15.0
Glucocorticoids	26.5
Thyroxin	19.4
Antihypertensives^a^	71.2
CNS active medications^a^	8.8
Heparin	1.7

*CNS* Central nervous system, *COPD* chronic obstructive pulmonary disease, *DME* durable medical equipment; *SD* standard deviation

^a^Osteoporosis medications include: bisphosphonates, calcitonin, teriparatide, raloxifene, denosumab. Antihypertensives include beta blockers, calcium channel blockers, renin-angiotensin system antagonists, antiadrenergic/sympatholytics, etc. CNS active medications include tryclic antidepressants, monoamine oxidase inhibitors, antipsychotic agents, lithium

During the 12- and 24-month follow-up periods, 3.0% and 5.4% of patients in the cohort had an incident osteoporotic fracture, respectively. The proportion of patients with an incident hip, vertebral or non-hip/non-vertebral fractures during the 24 months of follow-up were 1.4%, 1.6%, and 2.3% respectively.

Factors most strongly associated with fractures in 12 months of follow-up included older age, female sex, white race, history of fracture, history of falls, and comorbidities (osteoporosis, cardiovascular disease, COPD, ankylosing spondylitis, depression, and anxiety/mood disorders) (Table 2). Other factors associated with imminent risk of fracture included ambulance/life support, difficulty walking, use of durable medical equipment, and diminished physical function. The findings were largely consistent for factors associated with the risk of fracture during 24 months of follow-up.

**Table 2:** Factors associated with imminent risk of fracture in 12- and 24-month follow-ups

Factors	12-month risk		24-month risk	
	Hazard ratio	95% CI	Hazard ratio	95% CI
Demographic				
Age in years				
67–69	Reference		Reference	
70–74	1.22	1.17–1.26	1.22	1.19–1.26
75–79	1.69	1.63–1.76	1.73	1.68–1.77
80–84	2.29	2.21–2.37	2.38	2.32–2.44
85+	3.12	3.01–3.23	3.27	3.19–3.35
Male	0.55	0.54–0.56	0.54	0.53–0.55
Race				
While	Reference			
Black	0.43	0.41–0.45	0.42	0.41–0.44
Hispanic	0.70	0.66–0.75	0.72	0.69–0.75
Asian	0.66	0.62–0.71	0.66	0.63–0.69
Other	0.81	0.75–0.87	0.80	0.76—0.85
History of Fracture	2.46	2.41–2.52	2.21	2.17–2.25
History of Falls	1.18	1.14–1.21	1.16	1.14–1.19
Clinical conditions				
Osteoporosis	1.22	1.18–1.25	1.21	1.18–1.24
Cardiovascular disease	1.11	1.09–1.13	1.12	1.10–1.13
COPD	1.16	1.14–1.19	1.17	1.15–1.19
Ankylosing spondylitis	1.52	1.36–1.70	1.41	1.29–1.55
Depression	1.14	1.11–1.17	1.13	1.11–1.16
Mood or anxiety orders	1.07	1.03–1.11	1.10	1.07–1.13
Frailty markers				
Ambulance/life support	1.16	1.13–1.19	1.15	1.13–1.17
Difficulty walking	1.15	1.12–1.18	1.14	1.12–1.16
Use of DME	1.12	1.09–1.14	1.11	1.09–1.13
Concomitant medications				
Osteoporosis medications^a^	1.26	1.24–1.29	1.27	1.25–1.29
Glucocorticoids	1.13	1.11–1.15	1.13	1.12–1.15
Heparin	1.21	1.14–1.28	1.16	1.11–1.21
CNS active medications^a^	1.13	1.10–1.16	1.11	1.09–1.13

*CNS* central nervous system, *COPD* chronic obstructive pulmonary disease, *DME* durable medical equipment

^a^Osteoporosis medications include bisphosphonates, calcitonin, teriparatide, raloxifene, denosumab. CNS active medications include tricyclic antidepressants, monoamine oxidase inhibitors, antipsychotic agents, lithium

Table 3 shows the points that were assigned to each category of each of the factor evaluated as a fracture risk predictor with higher points indicating higher risk. Points differ for males and females in the study population and by follow-up time (12 months, 24 months). These points were used in the calculation of patient-level fracture risk scores; the distribution of scores in the study population is summarized in Supplemental Table 1. For both men and women, the distribution of patients by risk score was similar for the 12- and 24-month follow-up periods. The majority (69%) of men had 12-month risk scores between 16 and 30. Women tended to have higher risk scores, with 56% having 12-month scores of 26 to 50 and nearly one-quarter having12-month scores of 50 or higher. A similar shift was observed in comparing 24-month risk scores for men and women. The probability of fracture during the 12- and 24-month follow-up periods is shown for males and females at each risk score level and is provided in Supplemental Table 2.

**Table 3:** Points used in risk score calculation

Variable	Categories	Male		Female	
		One year	Two years	One year	Two years
Age					
	67–69	0	0	0	0
	70–74	4	4	4	4
	75–79	9	9	9	9
	80–84	14	14	14	14
	85+	25	25	25	25
White					
	No	0	0	0	0
	Yes	5	5	4	4
Black					
	No	6	5	14	13
	Yes	0	0	0	0
Hispanic					
	No	1	0	2	1
	Yes	0	1	0	0
Asian					
	No	4	5	4	3
	Yes	0	0	0	0
Previous fracture					
	No	0	0	0	0
	Yes	19	16	18	15
Osteoporosis		Not a Predictor	Not a Predictor		
	No			0	0
	Yes			5	4
CV disease		Not a Predictor			
	No		0	0	0
	Yes		3	2	2
COPD					
	No	0	0	0	0
	Yes	5	5	3	3
Anemia					Not a Predictor
	No	0	0	0	
	Yes	5	4	3	
Gastrointestinal disorders and toxicities		Not a Predictor	Not a Predictor	Not a Predictor	
	No				0
	Yes				2
Rheumatoid arthritis		Not a Predictor	Not a Predictor	Not a Predictor	
	No				0
	Yes				5
Depression		Not a Predictor	Not a Predictor		
	No			0	0
	Yes			4	3
Falls					
	No	0	0	0	0
	Yes	4	3	3	3
Ambulance life support					
	No	0	0	0	0
	Yes	5	4	3	2
Skin ulcer		Not a Predictor	Not a Predictor	Not a Predictor	
	No				4
	Yes				0
Difficulty walking				Not a Predictor	Not a Predictor
	No	0	0		
	Yes	5	5		
Use of DME		Not a Predictor	Not a Predictor		
	No			0	0
	Yes			3	2
Osteoporosis medications					
	No	0	0	0	0
	Yes	11	10	5	5
Glucocorticoids		Not a Predictor	Not a Predictor		
	No			0	0
	Yes			3	3

## Discussion

In this analysis of administrative claims from the 20% Medicare sample, the factors most strongly associated with imminent (12- and 24-month) risk of fracture included older age, female sex, white race, and history of fractures within the previous year. These results contribute to the understanding of imminent fracture risk by quantifying the real-world effect of these specific risk factors in a large population of men and women enrolled in Medicare. Individuals who were aged 80 years or older had two to three times the risk of fracture over the next 12 to 24 months as did individuals under age 70 years. Similarly, a history of fracture within 24 months of the index date doubled the risk of subsequent fracture in the near-term (within the next 12 and 24 months), and females were more than 80% more likely than males to experience a fracture in that interval. Among the comorbidities assessed as potential risk factors, codes for ankylosing spondylitis and osteoporosis were associated with the greatest increased risk (52% and 22%, respectively). History of falls, diminished physical function (e.g., difficulty walking, weakness, paralysis as indicated by diagnosis and procedure codes), and use of particular types of services (e.g., ambulance/life support, durable medical equipment), which may suggest frailty, were associated with a 12 to 16% increase in the risk of fracture, while the use of osteoporosis medications and heparin was associated with 26% and 21% greater risk, respectively. It is likely that patients prescribed osteoporosis medications during the pre-index period were at higher risk for fracture relative to those not prescribed osteoporosis therapy. It was beyond the scope of the current study to evaluate medication adherence in these patients, but the literature indicates that non-adherence is a significant challenge among patients with osteoporosis, and suboptimal adherence can significantly reduce the therapeutic benefits that patients derive from these medications [19, 20].

These findings are consistent with previous studies of factors that influence imminent risk of fracture. A recent case-control study of US men and women aged 50 years and older with commercial and Medicare supplemental insurance reported that in patients with osteoporosis and no recent fracture, falls, older age, poorer health status, comorbidities (psychosis, Alzheimer’s disease, central nervous system disease), and other potential fall risk factors were associated with increased risk of fracture in 12 and 24 months [12]. In an analysis of data obtained for Caucasian subjects in the Study of Osteoporotic Fractures (SOF) cohort, imminent risk of hip fracture was predicted by total hip T-score, non-vertebral fracture after age 50 years, and indicators of poor physical functioning. In this SOF population, age, total hip T-score, fracture after age 50 years, walking speed, Parkinson’s disease or stroke, smoking, and prior falls were independent risk factors for imminent non-vertebral fractures [14].

Together, the results of these studies indicate an important overlap between factors that influence imminent risk of fracture and those that influence risk in the long term (10 years), including age, race, indicators of bone health (osteoporosis diagnosis, bone mineral density), and history of fracture. When focusing specifically on fracture risk within 1–2 years, however, it is important to note that there are additional risk factors and to understand the dynamic nature of these risk factors as medical events occur or physical functioning changes. For example, it is well known that prior fractures at any site increase the risk of subsequent fracture, independent of BMD, and generally accepted that half of all patients who have had an osteoporotic fracture will go on to experience a subsequent fracture [21–30]. The scientific evidence demonstrating this risk relationship is so significant that the International Osteoporosis Foundation (IOF) has designated the prevention of subsequent fractures (i.e., secondary prevention) as the single most important opportunity to improve osteoporosis care and impact the trend of rapidly increasing fracture-related costs worldwide [31]. Analysis of data from a recent, large (18,872 individuals, 510,265 person-years follow-up) population-based cohort in Reykjavik, Iceland, however, suggests that the level of risk associated with prior fracture varies with both time since fracture and patient age [13]. In general, risk was highest immediately after the first fracture, and although risk declined over time, it never reached the level seen in the general population. Specifically, in the first year after the first major osteoporotic fracture, the risk of subsequent fracture was 2.7 (95% confidence interval: 2.4–3.0) times higher than the population risk. Ten years after the initial fracture, the risk ratio had fallen to 1.4 (1.2–1.6), and risk remained elevated for at least 15 years. Interestingly, this pattern of risk reduction over time appeared to be age-dependent and most evident in patients older than 60 years. These results illustrate how varying the time horizon used for fracture risk prediction can lead to different conclusions about the importance of key risk factors as the effect of these factors varies with time and within the context of other risk factors.

There is a wealth of epidemiologic evidence demonstrating strong associations between falls and fractures in the elderly [11]. In addition, not only has history of falls been associated with an increased risk of fracture in the near-term, but also current and previous studies have shown that factors that increase the risk of falls (e.g., poor physical functioning, mobility impairments, use of psychoactive medications) appear to influence fracture risk [12, 14]. These factors may also be quite dynamic, and changes in the patients fall history or fall-related risk profiles significantly impact their fracture risk as well.

In addition to identifying specific risk factors, the accumulating literature on imminent risk of fracture highlights the importance of ensuring that clinicians understand both the significance and dynamic nature of key risk factors for imminent risk of fracture. Routine monitoring for recent fractures, falls, or changes in physical functioning or mobility, incident diagnosis of conditions that affect coordination or balance, and the timely initiation of certain pharmacologic therapies may have the potential to reduce a patient’s imminent risk of fracture. The therapeutic benefit associated with early intervention following an initial fracture is expected to be significant in the elderly, and it seems reasonable to assume that the ability to routinely identify other significant predictors of imminent risk will provide an opportunity to more effectively manage at-risk patients [13]. Ensuring appropriate prescribing and use of osteoporosis medications may be challenging in the Medicare population, including among patients who have already sustained an osteoporotic fracture. One recent study, for example, found that only 30% of Medicare-enrolled patients (*N* = 145,185) who sustained a fracture between 2008 and 2011 filled a prescription for an osteoporosis medication during the 12 months post-fracture [32].

Our study has a number of strengths which enhance the generalizability and rigor of the results. The primary strength of our study is the availability of detailed clinical data for a large sample of elderly individuals, which should effectively generalize to the Medicare population overall since the study population was drawn from the 20% Medicare sample. Although osteoporosis and osteoporosis-related fractures disproportionately affect postmenopausal women, the inclusion of men in our study provides a more complete understanding of factors that influence imminent risk of fracture than a single-sex study would provide. To our knowledge, only one other study of US data has included males in evaluating predictors of imminent risk, and that study which included commercially insured individuals and Medicare enrollees aged 50 years and older did not identify sex as a significant risk predictor [12]. Access to detailed clinical and demographic data over 24 months of pre- and post-index periods allowed us to evaluate the role of a diverse set of potential risk factors on incident fracture in 12 and 24 months follow-up intervals. That said, some potential risk factors documented in previous studies (e.g., specific measures of mobility or physical functioning [grip strength, walking speed]) are not evaluable in administrative claims data. We also note that only outpatient medication fills were included, and medication use may have changed for individual patients during follow-up. However, these predictors were not defined in a time-varying manner in the models, so the impact of such changes is not captured in these results. Although the presence/absence of historical fracture was noted in the study database, our ability to characterize these prior fractures was limited since details on the timing and site of those prior fractures were not available. In addition, since it is challenging to identify incident fractures (our primary study outcome) in administrative claims data, we used a previously published claims-based algorithm [16] . This approach likely reduced but may not have eliminated all potential misclassification of this outcome. Finally, although patients may have died during follow-up, death was not considered as a competing risk in the multivariate models. Future studies may be able to not only build upon the current study but also address important limitations by including detailed data on the recency of prior fracture and treatment adherence, as well as considering death as a competing risk.

Our study shows that advancing age, female sex, recent prior fracture and falls, and specific comorbidities and medications were factors contributing to imminent risk of fracture among Medicare-aged individuals. While there is overlap between risk factors that shape imminent and long-term fracture risk, the dynamic nature of certain risk factors is particularly important for accurately estimating imminent risk and making appropriate decisions regarding treatment. For example, elderly patients who initiate osteoporosis medications soon after an initial fracture when the increased risk of subsequent fracture is highest will have the greatest opportunity to derive critical therapeutic benefit and potentially avoid a second fracture. Health care providers may also inform their choice of therapy with an assessment of imminent risk in a given patient, and, for example, may prescribe a bone-forming agent for rapid fracture risk reduction prior to antiresorptive therapy for a high-risk patient. Similarly, clinicians who routinely monitor their patients for changes in key imminent risk factors including recent falls or changes in fall risk factors, medical events (e.g., stroke), comorbidities, or medication regimens may have an opportunity to identify patients to receive therapy, change therapy, or improve their treatment compliance as patients’ risk of fracture increases. The timing of routine assessment must be considered, but clinicians may consider annual assessments for patients of average risk and more frequent assessment for patients with multiple risk factors or rapidly changing health. Clinician awareness of these risk factors and their dynamic nature may lead to improved osteoporosis care for elderly patients.

## Electronic supplementary material

ESM 1 (DOCX 47.1 kb)
