# Supplementary material for: Predictors of imminent risk of fracture in Medicare-enrolled men and women
Source: Arch Osteoporos. 2020 Aug 3;15(1):120. doi: 10.1007/s11657-020-00784-7 (PMC7399683; doi:10.1007/s11657-020-00784-7)
Supplement: Supplementary file 1 — (DOCX 47.1 kb) [file 11657_2020_784_MOESM1_ESM.docx]

Supplemental Table 1. Distribution of risk scores in study population

1. Males

| One Year | | | Two Years | | |
| --- | --- | --- | --- | --- | --- |
| Risk score | Percent of patients | Cumulative | Risk score | Percent of patients | Cumulative |
| 5.0 | 0.9 | 0.9 | 5.0 | 1.0 | 1.0 |
| 6-10 | 2.1 | 3.0 | 6-10 | 2.2 | 3.2 |
| 11-15 | 3.5 | 6.5 | 11-15 | 13.3 | 16.5 |
| 16-20 | 34.7 | 41.2 | 16-20 | 20.4 | 36.9 |
| 21-25 | 20.1 | 61.3 | 21-25 | 18.3 | 55.2 |
| 26-30 | 14.0 | 75.3 | 26-30 | 14.8 | 70.0 |
| 31-35 | 6.2 | 81.5 | 31-35 | 9.5 | 79.5 |
| 36-40 | 3.3 | 84.8 | 36-40 | 7.6 | 87.1 |
| 41-45 | 6.8 | 91.6 | 41-45 | 5.5 | 92.6 |
| 46-50 | 3.6 | 95.3 | 46-50 | 3.1 | 95.7 |
| 51-55 | 2.0 | 97.3 | 51-55 | 1.9 | 97.6 |
| 56-60 | 1.2 | 98.5 | 56-60 | 1.2 | 98.8 |
| 61-65 | 0.6 | 99.2 | 61-65 | 0.6 | 99.4 |
| 66-70 | 0.3 | 99.5 | 66-70 | 0.3 | 99.6 |
| 71-75 | 0.3 | 99.8 | 71-75 | 0.3 | 99.9 |
| 76-80 | 0.2 | 99.9 | 76-80 | 0.1 | 100.0 |
| 81-85 | 0.1 | 100.0 | 81-85 | 0.0 | 100.0 |
| 86-90 | 0.0 | 100.0 | 86-90 | 0.0 | 100.0 |
| 91-95 | 0.0 | 100.0 | 91-95 | 0.0 | 100.0 |

Supplemental Table 1. Distribution of risk scores in study population

1. Females

| One Year | | | Two Years | | |
| --- | --- | --- | --- | --- | --- |
| Risk score | Percent of patients | Cumulative | Risk score | Percent of patients | Cumulative |
| 5.0 | 0.0 | 0.0 | 5.0 | 0.0 | 0.0 |
| 6-10 | 1.4 | 1.4 | 6-10 | 0.6 | 0.6 |
| 11-15 | 1.5 | 2.9 | 11-15 | 1.6 | 2.2 |
| 16-20 | 2.0 | 5.0 | 16-20 | 1.9 | 4.1 |
| 21-25 | 7.4 | 12.3 | 21-25 | 7.8 | 11.9 |
| 26-30 | 14.9 | 27.2 | 26-30 | 13.7 | 25.6 |
| 31-35 | 16.5 | 43.7 | 31-35 | 16.8 | 42.3 |
| 36-40 | 14.9 | 58.7 | 36-40 | 15.5 | 57.8 |
| 41-45 | 9.5 | 68.2 | 41-45 | 11.0 | 68.8 |
| 46-50 | 8.6 | 76.8 | 46-50 | 9.4 | 78.2 |
| 51-55 | 9.3 | 86.1 | 51-55 | 9.5 | 87.7 |
| 56-60 | 5.4 | 91.5 | 56-60 | 5.3 | 93.0 |
| 61-65 | 2.8 | 94.3 | 61-65 | 2.7 | 95.7 |
| 66-70 | 2.0 | 96.3 | 66-70 | 1.8 | 97.5 |
| 71-75 | 1.5 | 97.8 | 71-75 | 1.3 | 98.8 |
| 76-80 | 1.0 | 98.8 | 76-80 | 0.8 | 99.6 |
| 81-85 | 0.7 | 99.5 | 81-85 | 0.3 | 99.9 |
| 86-90 | 0.4 | 99.8 | 86-90 | 0.1 | 100.0 |
| 91-95 | 0.1 | 100.0 | 91-95 | 0.0 | 100.0 |
| 96-100 | 0.01 |  | 96-100 | 0 | 100.0 |

Supplemental Table 2. One-Year and Two-Year Probability of Fracture by Risk Score

| Fracture Risk Score | Male | | Female | |
| --- | --- | --- | --- | --- |
|  | One Year | Two Years | One Year | Two Years |
| 4 | 0.003 | 0.006 | 0.006 | 0.011 |
| 5 | 0.003 | 0.007 | 0.006 | 0.011 |
| 6 | 0.004 | 0.007 | 0.007 | 0.012 |
| 7 | 0.004 | 0.007 | 0.007 | 0.012 |
| 8 | 0.004 | 0.008 | 0.007 | 0.013 |
| 9 | 0.004 | 0.008 | 0.008 | 0.014 |
| 10 | 0.004 | 0.009 | 0.008 | 0.014 |
| 11 | 0.005 | 0.009 | 0.008 | 0.015 |
| 12 | 0.005 | 0.010 | 0.009 | 0.016 |
| 13 | 0.005 | 0.011 | 0.009 | 0.017 |
| 14 | 0.006 | 0.011 | 0.009 | 0.017 |
| 15 | 0.006 | 0.012 | 0.010 | 0.018 |
| 16 | 0.006 | 0.013 | 0.010 | 0.019 |
| 17 | 0.007 | 0.013 | 0.011 | 0.020 |
| 18 | 0.007 | 0.014 | 0.011 | 0.021 |
| 19 | 0.007 | 0.015 | 0.012 | 0.022 |
| 20 | 0.008 | 0.016 | 0.012 | 0.023 |
| 21 | 0.008 | 0.017 | 0.013 | 0.024 |
| 22 | 0.009 | 0.018 | 0.014 | 0.025 |
| 23 | 0.009 | 0.019 | 0.014 | 0.027 |
| 24 | 0.010 | 0.020 | 0.015 | 0.028 |
| 25 | 0.010 | 0.021 | 0.016 | 0.029 |
| 26 | 0.011 | 0.022 | 0.016 | 0.031 |
| 27 | 0.012 | 0.024 | 0.017 | 0.032 |
| 28 | 0.012 | 0.025 | 0.018 | 0.034 |
| 29 | 0.013 | 0.027 | 0.019 | 0.036 |
| 30 | 0.014 | 0.028 | 0.020 | 0.037 |
| 31 | 0.015 | 0.030 | 0.020 | 0.039 |
| 32 | 0.016 | 0.031 | 0.021 | 0.041 |
| 33 | 0.017 | 0.033 | 0.022 | 0.043 |
| 34 | 0.018 | 0.035 | 0.023 | 0.045 |
| 35 | 0.019 | 0.037 | 0.024 | 0.047 |
| 36 | 0.020 | 0.040 | 0.026 | 0.049 |
| 37 | 0.021 | 0.042 | 0.027 | 0.052 |
| 38 | 0.022 | 0.044 | 0.028 | 0.054 |
| 39 | 0.023 | 0.047 | 0.029 | 0.057 |

Supplemental Table 2. One-Year and Two-Year Probability of Fracture by Risk Score (continued)

| Fracture Risk Score | Male | | Female | |
| --- | --- | --- | --- | --- |
|  | One Year | Two Years | One Year | Two Years |
| 40 | 0.025 | 0.050 | 0.031 | 0.060 |
| 41 | 0.026 | 0.053 | 0.032 | 0.063 |
| 42 | 0.028 | 0.056 | 0.034 | 0.066 |
| 43 | 0.029 | 0.059 | 0.035 | 0.069 |
| 44 | 0.031 | 0.062 | 0.037 | 0.072 |
| 45 | 0.033 | 0.066 | 0.038 | 0.075 |
| 46 | 0.035 | 0.070 | 0.040 | 0.079 |
| 47 | 0.037 | 0.074 | 0.042 | 0.083 |
| 48 | 0.039 | 0.078 | 0.044 | 0.087 |
| 49 | 0.041 | 0.083 | 0.046 | 0.091 |
| 50 | 0.044 | 0.087 | 0.048 | 0.095 |
| 51 | 0.046 | 0.092 | 0.050 | 0.100 |
| 52 | 0.049 | 0.098 | 0.052 | 0.104 |
| 53 | 0.052 | 0.103 | 0.055 | 0.109 |
| 54 | 0.055 | 0.109 | 0.057 | 0.114 |
| 55 | 0.058 | 0.115 | 0.060 | 0.120 |
| 56 | 0.061 | 0.122 | 0.063 | 0.125 |
| 57 | 0.065 | 0.128 | 0.065 | 0.131 |
| 58 | 0.068 | 0.136 | 0.068 | 0.137 |
| 59 | 0.072 | 0.143 | 0.071 | 0.143 |
| 60 | 0.076 | 0.151 | 0.075 | 0.150 |
| 61 | 0.081 | 0.159 | 0.078 | 0.156 |
| 62 | 0.085 | 0.168 | 0.081 | 0.164 |
| 63 | 0.090 | 0.177 | 0.085 | 0.171 |
| 64 | 0.095 | 0.187 | 0.089 | 0.179 |
| 65 | 0.101 | 0.197 | 0.093 | 0.187 |
| 66 | 0.106 | 0.207 | 0.097 | 0.195 |
| 67 | 0.112 | 0.218 | 0.101 | 0.204 |
| 68 | 0.119 | 0.230 | 0.106 | 0.212 |
| 69 | 0.125 | 0.242 | 0.110 | 0.222 |
| 70 | 0.132 | 0.254 | 0.115 | 0.231 |
| 71 | 0.139 | 0.267 | 0.120 | 0.241 |
| 72 | 0.147 | 0.281 | 0.125 | 0.252 |
| 73 | 0.155 | 0.295 | 0.131 | 0.262 |
| 74 | 0.163 | 0.310 | 0.137 | 0.273 |
| 75 | 0.172 | 0.325 | 0.142 | 0.285 |

Supplemental Table 2. One-Year and Two-Year Probability of Fracture by Risk Score (continued)

| Fracture Risk Score | Male | | Female | |
| --- | --- | --- | --- | --- |
|  | One Year | Two Years | One Year | Two Years |
| 76 | 0.182 | 0.341 | 0.149 | 0.297 |
| 77 | 0.191 | 0.357 | 0.155 | 0.309 |
| 78 | 0.201 | 0.374 | 0.162 | 0.321 |
| 79 | 0.212 | 0.391 | 0.168 | 0.334 |
| 80 | 0.223 | 0.409 | 0.176 | 0.348 |
| 81 | 0.235 | 0.427 | 0.183 | 0.361 |
| 82 | 0.247 | 0.446 | 0.191 | 0.375 |
| 83 | 0.259 | 0.466 | 0.199 | 0.390 |
| 84 | 0.272 | 0.485 | 0.207 | 0.404 |
| 85 | 0.286 | 0.505 | 0.215 | 0.420 |
| 86 | 0.300 | 0.526 | 0.224 | 0.435 |
| 87 | 0.315 | 0.547 | 0.233 | 0.451 |
| 88 | 0.330 | 0.568 | 0.243 | 0.467 |
| 89 | 0.346 | 0.589 | 0.252 | 0.483 |
| 90 | 0.362 | 0.610 | 0.263 | 0.500 |
| 91 | 0.379 | 0.632 | 0.273 | 0.517 |
| 92 | 0.396 | 0.653 | 0.284 | 0.534 |
| 93 | 0.414 | 0.675 | 0.295 | 0.551 |
| 94 | 0.433 | 0.696 | 0.306 | 0.569 |
| 95 | 0.451 | 0.717 | 0.318 | 0.586 |
| 96 | 0.471 | 0.737 | 0.330 | 0.604 |
| 97 | 0.490 | 0.758 | 0.342 | 0.622 |
| 98 | 0.510 | 0.777 | 0.355 | 0.640 |
| 99 | 0.531 | 0.797 | 0.368 | 0.657 |
| 100 | 0.551 | 0.815 | 0.382 | 0.675 |
| 101 | 0.572 | 0.833 | 0.395 | 0.693 |
